# Association between blood metabolites and chronic respiratory diseases: A Mendelian randomization study

**DOI:** 10.1097/MD.0000000000048317

**Published:** 2026-04-24

**Authors:** Peng Gao, Dongqing Ye, Qinglong Li, Lu Bai, Xihai Xu, Xinyu Fang

**Affiliations:** a School of Public Health, Anhui University of Science and Technology, Huainan, Anhui Province, China; b Inflammation and Immune Mediated Diseases Laboratory of Anhui Province, Hefei, Anhui Province, China; c Health Management Center, The First Affiliated Hospital of Anhui Medical University, Hefei, Anhui Province, China; d Joint Research Center for Occupational Medicine and Health of IHM, Anhui University of Science and Technology, Huainan, Anhui Province, China; e Epidemiology and Health Statistics, School of Public Health, Anhui Medical University, Hefei, Anhui Province, China.

**Keywords:** asthma, COPD, idiopathic pulmonary fibrosis, Mendelian randomization, pneumoconiosis, sarcoidosis, serum metabolites

## Abstract

Chronic respiratory diseases (CRDs) are a growing global health concern. Emerging evidence implicates circulating metabolites in their development and progression, but definitive causal insights into how metabolic patterns influence disease pathways remain limited. We conducted a two-sample Mendelian randomization (MR) analysis to evaluate potential causal effects of 1400 serum metabolites on 5 major CRDs: chronic obstructive pulmonary disease (COPD), asthma, idiopathic pulmonary fibrosis (IPF), sarcoidosis, and pneumoconiosis. Each disease was analyzed separately; no composite CRD outcome was constructed. The inverse-variance weighted method was the primary approach, complemented by MR-Egger, weighted median, and MR-PRESSO for outlier detection and correction. Robustness was examined using sensitivity analyses, including Cochran *Q* for heterogeneity, MR-Egger intercept for directional pleiotropy, the MR-PRESSO global test, and leave-one-out analyses. MR analyses identified significant causal associations between multiple metabolites/metabolite ratios and the 5 CRDs, revealing distinct metabolic profiles for each disease. For COPD, we found 54 potentially causal metabolites (30 risk, 24 protective). Asthma showed 36 associations (13 risk, 20 protective). IPF had 39 (22 risk, 17 protective). Sarcoidosis exhibited the broadest signature with 69 associations (24 risk, 45 protective). Pneumoconiosis showed 53 (25 risk, 28 protective). Most signals were disease-specific; only a small subset overlapped across at least 2 diseases (e.g., four shared between COPD and sarcoidosis, two between pneumoconiosis and asthma), suggesting partially shared pathways. No metabolite displayed consistent associations across all 5 diseases. Circulating metabolites exhibit protective or detrimental causal effects on COPD, asthma, IPF, sarcoidosis, and pneumoconiosis. Effects are heterogeneous and largely disease-specific, with limited overlap across conditions, indicating predominantly distinct etiologic pathways and offering mechanistic insights that may inform risk stratification and target prioritization. Further validation using integrated multi-omics and experimental models is warranted to refine mechanisms and assess translational potential.

## 1. Introduction

Chronic respiratory diseases (CRDs) comprise a range of pathological disorders mainly impacting the lungs and airways. This group includes chronic obstructive pulmonary disease (COPD), asthma, pneumoconiosis, interstitial lung disease, and pulmonary sarcoidosis. According to 2019 global epidemiological data, CRDs affected an estimated 454.6 million individuals worldwide and saw approximately 77.6 million new cases. Alarmingly, this incidence reflects a 49.0% increase (95% CI: 42.1%–55.6%) relative to 1990 levels. Ranking as the world’s third most common cause of death, CRDs were responsible for 4 million fatalities (95% UI: 3.6–4.3 million) and imposed substantial healthcare costs, representing a major socioeconomic burden.^[1]^ Despite the absence of curative treatments, contemporary therapeutic interventions demonstrate significant clinical value in symptom management and quality-of-life enhancement. Evidence-based strategies can effectively mitigate adverse outcomes linked to elevated morbidity rates, frequent healthcare utilization, disability progression, and mortality risk. Current clinical paradigms emphasize comprehensive disease management protocols targeting exacerbation prevention and complication reduction through optimized pharmacotherapy and multidisciplinary care approaches.

Although the origins of CRDs are not fully understood, both genetic and environmental elements play major roles in their development. Furthermore, mounting research associates changes in circulating metabolite patterns with CRDs.^[2,3]^ These small molecules serve as intermediates or final products within biochemical pathways. Metabolite concentrations are influenced by multiple determinants, including genetics, diet, lifestyle, gut microbiome composition, and disease states. Notably, these bioactive compounds exhibit bidirectional interactions with disease pathogenesis - while influencing disease susceptibility through modulation of physiological processes, they simultaneously serve as therapeutic targets for intervention strategies.^[4–6]^ Recent studies indicate notable links involving serum levels of specific lipid mediators and hormones, such as oleoylethanolamide, dehydroepiandrosterone sulfate, and cortisone, and the severity of asthma.^[7]^ Significantly, serum uric acid, a measurable marker of oxidative stress, may serve as a biomarker for asthma severity assessment.^[8]^ Analyses comparing patients with COPD to healthy controls showed markedly lower serum concentrations of 1-methylnicotinamide, creatinine, and lactate.^[9]^ Particularly, glutamylphenylalanine was identified as a potential biomarker for COPD exacerbation events, while sphingolipid profiles showed correlations with pulmonary function parameters.^[10,11]^ Peng plasma analysis of 61 stage I coal workers’ pneumoconiosis (CWP) patients, 84 dust-exposed workers, and 51 healthy controls revealed 3 distinct metabolic discriminators: lysophosphatidylinositol, lysophosphatidylcholine, and serum bilirubin.^[12]^ Although existing studies have revealed associations between microbial metabolites and CRDs, the causal relationship remains to be established. It is imperative to elucidate whether gut microbiota actively contributes to CRDs pathogenesis through causal mechanisms or merely represents a downstream manifestation of shared risk factors.

Mendelian randomization (MR) is an emerging statistical methodology designed to infer causal relationships by emulating the design of randomized controlled trials (RCTs).^[13]^ This method exploits the fundamental principle of random genetic variant assortment during gamete formation, resembling the randomization process in clinical trials. Within the MR framework, single nucleotide polymorphisms (SNPs) function as instrumental variables to estimate causal relationships between exposures and outcomes. By leveraging the inherent randomness of genetic inheritance, this method effectively mitigates confounding bias that frequently plagues observational studies. Furthermore, the unidirectional nature of genetic influence (from genotype to phenotype) inherently eliminates potential reverse causation bias, as disease progression cannot retroactively alter germline genetic variants.^[14]^

We performed an extensive bidirectional MR investigation to explore connections linking 1400 circulating metabolites with 5 prevalent chronic respiratory conditions: asthma, COPD, idiopathic pulmonary fibrosis (IPF), pneumoconiosis, and sarcoidosis. Our results clarify metabolites’ potential involvement in the underlying mechanisms of these diseases and could guide novel preventive or therapeutic approaches.

## 2. Methods

### 2.1. Study design

**Figure 1.**
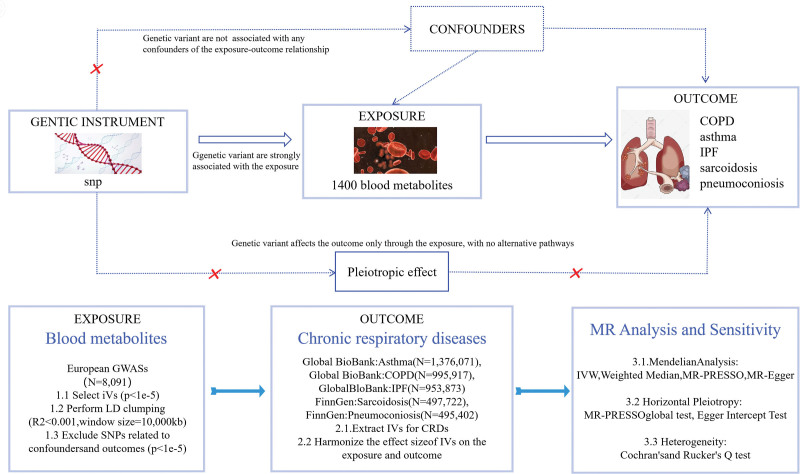
Project implementation flowchart. The flowchart summarizes data sources, instrumental variable selection, LD clumping, primary MR methods (IVW, MR-Egger, weighted median), MR-PRESSO outlier detection, and sensitivity analyses. GWAS = genome-wide association study, MR = Mendelian randomization; IVs = instrumental variables, LD = linkage disequilibrium, SNP = single nucleotide polymorphism, IVW = inverse-variance weighted, MR-PRESSO = Mendelian randomization pleiotropy residual sum and outlier, COPD = chronic obstructive pulmonary disease, IPF = idiopathic pulmonary fibrosis.

We employed a two-sample MR framework to systematically investigate causal links between 1400 circulating metabolites and 5 chronic respiratory conditions: asthma, COPD, IPF, pneumoconiosis, and sarcoidosis. Bidirectional MR analyses were implemented to mitigate reverse causation by evaluating both forward and reverse causal directions. Our methodology strictly satisfied the core MR assumptions: instrumental variables (IVs) demonstrated robust associations with target exposures; IVs remained independent of confounding variables; and IVs affected outcomes exclusively via exposure pathways. This methodological rigor ensures valid causal inference, offering crucial insights into metabolites’ pathogenic roles in respiratory diseases. The study flowchart is shown in Figure 1.

### 2.2. Data sources

Genetic information for blood metabolites originated from the Canadian Longitudinal Study on Aging cohort, encompassing 8091 individuals.^[15]^ This investigation examined 1091 unique metabolites and 309 metabolite ratios. Rigorous quality control protocols were implemented, retaining only SNPs meeting the following criteria: minor allele frequency exceeding 0.1%, imputation quality scores >0.3, and missingness rates <0.1%. This stringent filtering resulted in approximately 15.4 million high-quality SNPs (reference genome v38) for downstream Genome-wide association studies (GWAS), minimizing artifacts from poorly imputed variants and strengthening analytical robustness.^[15]^ Chronic respiratory disease GWAS data, specifically for COPD, asthma, and IPF, were sourced from a global biobank meta-analysis initiative (GBMI) meta-analysis. This collaborative network integrates 23 biobanks across 4 continents, providing genetic and electronic health records from over 2.2 million consenting individuals.^[16]^ Case-control counts were as follows: COPD: 58,559 cases versus 937,858 controls, Asthma: 121,940 cases versus 1254,131 controls, IPF: 6257 cases versus 947,616 controls. Genetic associations for sarcoidosis (5411 cases; 492,311 controls) and pneumoconiosis (807 cases; 494,595 controls) originated from the latest FinnGen Biobank release.^[17]^ Comprehensive sample characteristics appear in Table 1.

**Table 1:** Characteristics of the GWAS used for analyses.

Trait	Data type	N_cases	N_controls	Ethnicity	Accession numbers	PubMed ID
Blood metabolites	Exposure	1400		European	GCST90199621-90201020	36635386
COPD	Outcome	58,559	937,358	European	GCST90399694	36777996
Asthma	Outcome	121,940	1254,131	European	GCST90399686	36777996
IPF	Outcome	6257	947,616	European	GCST90399720	36777996
Sarcoidosis	Outcome	5411	492,311	European	Sarcoidosis	36653562
Pneumoconiosis	Outcome	807	494,595	European	Pneumoconiosis	36653562

COPD = chronic obstructive pulmonary disease, IPF = idiopathic pulmonary fibrosis.

### 2.3. Instrument variables selection

To ensure causal inference validity between circulating metabolites and CRDs, we implemented stringent quality control procedures for IV selection, satisfying core MR assumptions. Given limited SNP availability, initial metabolite-associated variants (*P* < 1 × 10^−5^) underwent linkage disequilibrium clumping (*r*^2^ < 0.001; 10,000 kb window). Instrument strength was evaluated via *F*-statistic calculation (β^2^/SE^2^), excluding variants with *F* < 10 to mitigate weak instrument bias.^[18]^ To prevent horizontal pleiotropy, outcome-associated SNPs (*P* < 1 × 10^−5^) were systematically removed. Final candidate variants underwent PhenoScanner screening to eliminate associations with potential confounders (Tables S1–S5, Supplemental Digital Content, https://links.lww.com/MD/R744, https://links.lww.com/MD/R745, https://links.lww.com/MD/R746, https://links.lww.com/MD/R747).

### 2.4. Statistical analysis

Using curated IVs, we conducted bidirectional MR analyses to evaluate causal links between circulating metabolites and 5 CRD. Analyses were performed in R (v4.1.2) using the Two Sample MR and MR-PRESSO packages. Five complementary methods were implemented: Random-effects inverse-variance weighted (IVW) as the principal method, MR-Egger regression, Weighted median, Simple mode, and Weighted mode. The random-effects IVW approach formed our primary analytical foundation. When IV assumptions hold, this method combines Wald ratio-derived causal estimates from individual SNPs using meta-analytical techniques, yielding robust effect estimates for exposure-outcome relationships.^[19]^ While MR-Egger regression relaxes the zero-mean pleiotropy assumption, it generally suffers from reduced statistical power.^[20]^ The weighted median method provides consistent causal estimates when valid instruments contribute ≥50% of the weighting.^[21]^ For pleiotropy-robust model-based estimation, we employed the simple mode approach,^[22]^ whereas the weighted mode technique displays enhanced sensitivity to clustered instrument strength.^[23]^ We evaluated heterogeneity using the Cochran *Q* statistic (IVW method) and Rucker Q statistic (MR-Egger), considering *P* > .05 indicative of nonsignificant heterogeneity.^[24]^ Potential horizontal pleiotropy was assessed via MR-Egger intercept testing (*P* > .05, implying absence of detectable pleiotropy).^[25]^ Leave-one-out sensitivity analyses further determined whether individual SNPs disproportionately influenced observed metabolite-CRD causal relationships.^[19]^

### 2.5. Ethics statement

This MR study used publicly available, de-identified summary statistics from previously published GWAS. No new individual-level data were collected, and no human participants were recruited; therefore, additional ethical approval and informed consent were not required. The original GWAS obtained ethics approval and informed consent from participants, as reported in their publications. This study adhered to the Declaration of Helsinki.

## 3. Results

### 3.1. Selection of instrumental variables

Our analysis of 1400 blood metabolites utilized instrumental SNPs ranging from 9 to 38 per metabolite, with corresponding *F*-statistic values between 11.5 and 1421.7 (Tables S1–S5, Supplemental Digital Content, https://links.lww.com/MD/R744, https://links.lww.com/MD/R745, https://links.lww.com/MD/R746, https://links.lww.com/MD/R747). Notably, all SNPs demonstrated strong instrument strength (*F*-statistic > 10), effectively mitigating potential bias from weak instrumental variables and supporting the reliability of our causal inference.

### 3.2. Causal relationships between blood metabolites and COPD

**Figure 2.**
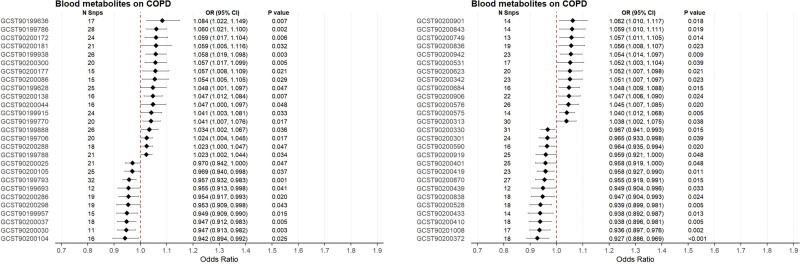
Mendelian randomization analysis of blood metabolites on COPD risk. CI = confidence interval, COPD = chronic obstructive pulmonary disease, OR = odds ratio, SNP = single nucleotide polymorphism.

Following the exclusion of SNPs strongly associated with smoking, MR analysis of 1400 blood metabolites for their causal effects on COPD was performed (Tables S6, Supplemental Digital Content, https://links.lww.com/MD/R748). Initial IVW estimates identified 69 metabolites nominally associated with COPD, including 38 positively correlated and 31 negatively correlated with COPD risk. After rigorous quality control involving heterogeneity tests (Cochran *Q P* > .05), horizontal pleiotropy evaluation, and reverse causality validation, 54 metabolites retained potential causal associations with COPD: 30 exhibiting risk-increasing effects and 24 demonstrating protective effects (Fig. 2). Sensitivity analyses confirmed the robustness of these associations, with leave-one-out analyses, scatter plots, and funnel plots provided in Table S6, Supplemental Digital Content, https://links.lww.com/MD/R748 and Figure S1, Supplemental Digital Content, https://links.lww.com/MD/R743.

### 3.3. Causal relationships between blood metabolites and asthma

**Figure 3.**
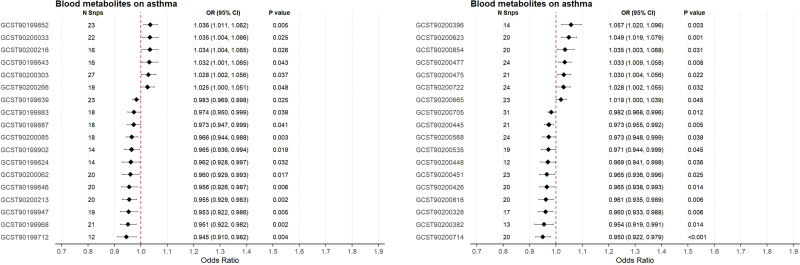
Mendelian randomization analysis of blood metabolites on asthma risk. CI = confidence interval, OR = odds ratio, SNP = single nucleotide polymorphism.

The IVW method identified 36 metabolites exhibiting putative causal associations with asthma, comprising 13 risk-increasing metabolites and 23 protective metabolites (Fig. 3). Sensitivity analyses confirmed the robustness of these associations: Cochran *Q* test revealed no significant heterogeneity (*P* > .05), MR-Egger regression intercept analysis demonstrated no evidence of horizontal pleiotropy (intercept *P* > .05), and reverse causality was excluded through directional validation. Leave-one-out sensitivity analysis further indicated that no single SNP disproportionately influenced the overall effect estimates (Table S7, Supplemental Digital Content, https://links.lww.com/MD/R748). Scatter plots visually reinforced the consistency of instrumental variable effects across exposure-outcome relationships, while funnel plot symmetry suggested minimal directional pleiotropy: though interpretation should consider the limited SNP number. Forest plots detailing individual SNP contributions to pooled effects are provided in Figure S2, Supplemental Digital Content, https://links.lww.com/MD/R743.

### 3.4. Causal relationships between blood metabolites and IPF

**Figure 4.**
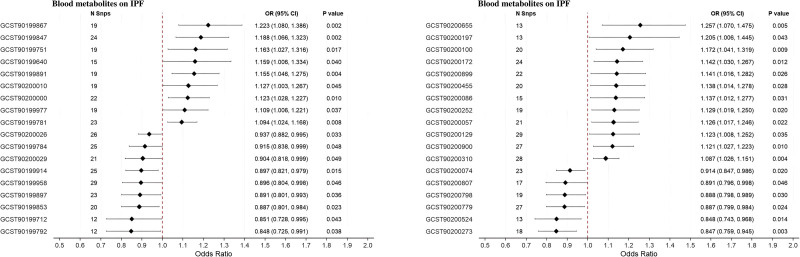
Mendelian randomization analysis of blood metabolites on IPF risk. CI = confidence interval, OR = odds ratio, SNP = single nucleotide polymorphism.

The IVW analysis identified 39 blood metabolites exhibiting potential causal relationships with IPF (Fig. 4). Among these, 22 metabolites demonstrated risk-increasing effects, exemplified by gamma-glutamylmethionine (odds ratio [OR] = 1.16, 95% CI: 1.03–1.32; *P* = .016), while 17 metabolites showed protective associations, including 2-methoxyhydroquinone sulfate (OR = 0.85, 95% CI: 0.76–0.95; *P* = .003). Robustness of these associations was supported by the Cochran *Q* test (*P* > .05), indicating no significant heterogeneity, and MR-Egger intercept analysis revealing no evidence of horizontal pleiotropy (intercept *P* > .05). (Table S8, Supplemental Digital Content, https://links.lww.com/MD/R748). Furthermore, leave-one-out sensitivity analysis confirmed that no single instrumental SNP disproportionately influenced the causal estimates (Figure S3, Supplemental Digital Content, https://links.lww.com/MD/R743).

### 3.5. Causal relationships between blood metabolites and sarcoidosis

**Figure 5.**
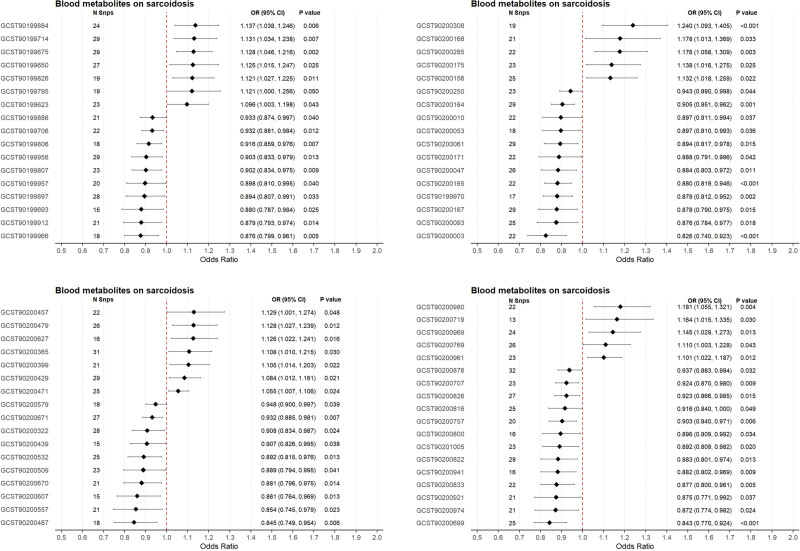
Mendelian randomization analysis of blood metabolites on sarcoidosis risk. CI = confidence interval, OR = odds ratio, SNP = single nucleotide polymorphism.

Under a comprehensive sensitivity analysis framework encompassing heterogeneity tests (Cochran *Q P* > .05), horizontal pleiotropy assessments (MR-Egger intercept *P* > .05), and reverse causality validation (Table S9, Supplemental Digital Content, https://links.lww.com/MD/R748 and Figure S4, Supplemental Digital Content, https://links.lww.com/MD/R743), IVW analysis revealed 69 metabolites exhibiting causal associations with sarcoidosis risk (Fig. 5). The analysis identified 24 risk-increasing metabolites, most notably 3-hydroxyisobutyrate (OR = 1.24, 95% CI: 1.09–1.41; *P* = 1.3 × 10^−4^), alongside 45 protective metabolites, with p-cresol glucuronide demonstrating the strongest inverse association (OR = 0.83, 95% CI: 0.74–0.92; *P* = 7.2 × 10^−4^).

### 3.6. Causal relationships between blood metabolites and pneumoconiosis

**Figure 6.**
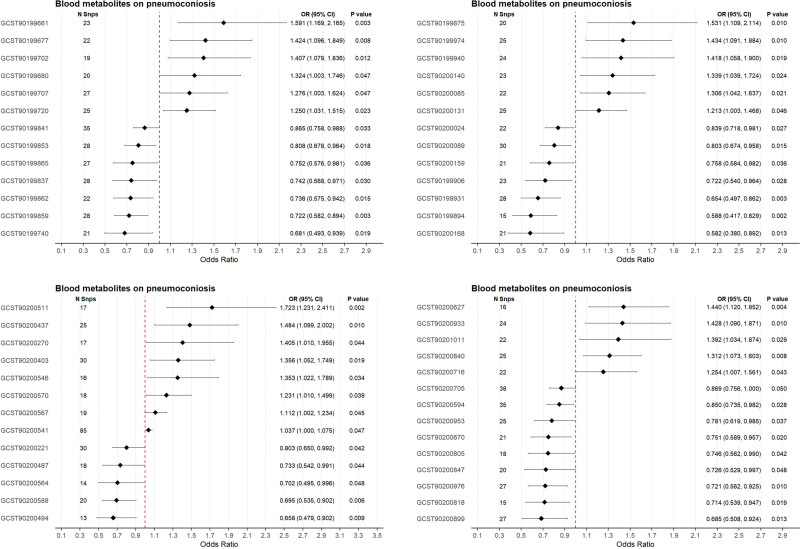
Mendelian randomization analysis of blood metabolites on pneumoconiosis risk. For each outcome, one representative exposure is shown to illustrate the consistency and robustness of the Mendelian randomization analyses. Forest plots, scatter plots, funnel plots, and leave-one-out sensitivity analyses are shown for selected exposures. CI = confidence interval, OR = odds ratio, SNP = single nucleotide polymorphism.

The IVW analysis identified 53 blood metabolites with potential causal associations with pneumoconiosis (Fig. 6). Among these, 25 metabolites exhibited risk-enhancing effects, maleate (OR = 1.59, 95% CI: 1.17–2.17; *P* = .003), while 28 metabolites demonstrated protective associations, including hydroxyasparagine (OR = 0.58, 95% CI: 0.38–0.89; *P* = .013). Robustness of these relationships was confirmed through multiple sensitivity analyses: Cochran *Q* test revealed no significant heterogeneity (*P* > .05), MR-Egger regression showed no detectable horizontal pleiotropy, and leave-one-out sensitivity analysis confirmed the absence of influential SNPs disproportionately driving causal estimates (Table S10, Supplemental Digital Content, https://links.lww.com/MD/R748 and Figure S5, Supplemental Digital Content, https://links.lww.com/MD/R743).

## 4. Discussion

RCTs remain the gold standard for epidemiological causal inference; their rigorous methodological demands and substantial expenses frequently limit broad application. Traditional observational studies often produce ambiguous causal interpretations owing to residual confounding and reverse causality.^[26]^ MR addresses these limitations by utilizing genetic variants’ random assortment and instrumental variable independence to enhance causal inference validity.^[27]^ Our MR investigation revealed distinctive circulating metabolite signatures associated with 5 chronic respiratory conditions: COPD: 54 metabolites, Asthma: 36 metabolites, IPF: 39 metabolites, Sarcoidosis: 69 metabolites, Pneumoconiosis: 53 metabolites. To contextualize the clinical relevance of our findings, we interpreted effect sizes in addition to statistical significance. We categorized metabolite: disease associations by the estimate (per SD increase in metabolite level) with supporting consistency across sensitivity analyses. In our study, we considered OR = 1.01–1.10 as modest, 1.10–1.20 as moderate, and >1.20 as strong effects. Under this framework, several signals for IPF (e.g., tridecenedioate (C13:1-DC) levels, N-lactoyl isoleucine levels, and X-25828 levels) fell into the strong range. For sarcoidosis, 3-hydroxyisobutyrate levels showed a strong risk association. For Asthma, pneumoconiosis, and COPD, most associations were modest, with a smaller subset reaching moderate effects. Although modest effects at the individual-metabolite level may appear small, their population impact can be substantial given the high prevalence of CRDs and the modifiable nature of metabolite exposures through diet, microbiome, or pharmacologic interventions.

These disease-specific metabolic patterns illuminate potential etiological mechanisms warranting further validation through integrated multi-omics investigations and functional analyses. Among sarcoidosis-related metabolites, 3-hydroxyisobutyrate (3-HIB), a valine catabolic intermediate, showed a relatively strong association (per–SD OR = 1.24, 95% CI 1.09–1.41). 3-HIB is produced by skeletal muscle and other tissues and acts as a paracrine signal to enhance endothelial fatty-acid transport and cellular lipid uptake, thereby increasing mitochondrial substrate load and oxidative stress, which may foster a pro-inflammatory microenvironment relevant to granuloma formation.^[28]^ N-lactoyl isoleucine is part of the N-lactoyl–amino acid family formed via CNDP2‑dependent reverse proteolysis from lactate and amino acids; these metabolites rise with increased glycolytic flux (e.g., exercise) and relate to immune/metabolic signaling, suggesting a pro‑inflammatory milieu relevant to IPF.^[29]^ The study identified kynurenine levels, imidazole propionate levels, and arachidonoylcholine levels as potential risk factors for COPD, while arginine levels were found to be protective factors. COPD features amplified local and systemic inflammation, characterized by recruitment and activation of diverse immune cells (particularly neutrophils).^[30]^ Individuals with COPD demonstrate increased concentrations of multiple inflammatory mediators such as chemokines, cytokines, and reactive oxygen species (ROS).^[31,32]^ Prior studies have demonstrated a significant elevation in circulating Tc17/IFN-γ cell populations among COPD patients, with levels positively correlating with disease severity.^[33]^ Building upon this evidence and considering that IFN-γ is known to upregulate indoleamine 2,3-dioxygenase (IDO) expression across various cell types^[34]^ - an enzyme critical for tryptophan (Trp) catabolism into kynurenine (Kyn) derivatives - we postulate that COPD pathogenesis may involve dysregulation of the Trp metabolic pathway through IDO overexpression. Arginine serves as the primary substrate for endogenous nitric oxide (NO) synthesis. As a pleiotropic signaling molecule, NO plays pivotal roles in both physiological and pathological processes of the respiratory system.^[35]^ Emerging evidence suggests that arginine may attenuate ROS production.^[36]^ ROS can disrupt cellular oxidative homeostasis and endoplasmic reticulum function. Excessive ROS generation may trigger an imbalance between endogenous proteases and antiproteases, thereby exacerbating pulmonary tissue damage. Furthermore, elevated ROS levels induce DNA damage, lipid peroxidation, and protein carbonylation in airway epithelial cells, potentially contributing to COPD progression. Our analysis identified elevated concentrations of phenylalanine, arachidonoylcholine, isoleucine, and maleate correlating with higher incidence risks for asthma, IPF, sarcoidosis, and pneumoconiosis. Arginine metabolism’s fundamental role in asthma pathogenesis is well-documented. This pivotal amino acid regulates multiple immunological and physiological processes essential for: T lymphocyte survival/activation,^[37]^ Macrophage polarization,^[38]^ Vasomotor tone regulation,^[39]^ Bronchodilation and bronchoconstriction suppression. Mechanistically, arginine functions as the principal substrate for inducible NO• synthase. Enzymatic conversion to citrulline generates NO•, a critical free radical signaling molecule.^[40]^ Notably, the alternative metabolic pathway involving arginase-mediated conversion to ornithine has also been implicated in asthma pathogenesis, as evidenced by both allergic animal models and clinical observations in human asthmatics.^[41,42]^

This investigation exhibits key methodological strengths. First, our bidirectional MR framework, utilizing genetic variants as instrumental variables, revealed potential causal links between circulating metabolites and 5 chronic respiratory conditions while minimizing confounding biases inherent in observational research. Second, compared to conventional RCTs, our genetic-level approach maintains scientific rigor through Mendel’s law of independent assortment while offering superior temporal and cost efficiencies. Third, we implemented comprehensive sensitivity analyses, including weighted median, MR-Egger, and MR-PRESSO methods to systematically address potential heterogeneity and pleiotropic effects.

This study has 3 important limitations that merit careful consideration. First, the exclusive focus on European-ancestry populations may limit the generalizability of our findings. To address this, we are actively collaborating with initiatives like the Global Biobank Meta-analysis Consortium to incorporate diverse cohorts from East Asian and African populations in future analyses. Second, while our study encompassed 5 CRDs, including pneumoconiosis, the relatively limited sample size for pneumoconiosis cases may have reduced the statistical power of our analyses. Expanding registry linkages will be crucial to overcoming this limitation in subsequent research. Finally, although our MR framework provides robust genetic evidence, translating these findings into clinical applications will require additional mechanistic validation. This includes in vitro studies using alveolar macrophage models and longitudinal cohort studies to evaluate the therapeutic potential of polyunsaturated fatty-acid interventions.

## 5. Conclusions

This two-sample MR study suggests that circulating metabolites have potential causal effects on 5 CRD, exhibiting disease-specific patterns with limited overlap across conditions. These findings prioritize candidate metabolites for risk stratification and mechanistic validation, warranting further confirmation through integrated multi-omics and experimental studies to refine target selection and assess translational potential.

## Author contributions

**Methodology:** Peng Gao.

**Conceptualization:** Dongqing Ye, Xinyu Fang.

**Supervision:** Dongqing Ye.

**Formal analysis:** Qinglong Li, Lu Bai.

**Data curation:** Xihai Xu.

**Writing – original draft:** Peng Gao.

**Writing – review & editing:** Xinyu Fang.

## Supplementary Material
