# Supplementary material for: Association between blood metabolites and chronic respiratory diseases: A Mendelian randomization study
Source: Medicine (Baltimore). 2026 Apr 24;105(17):e48317. doi: 10.1097/MD.0000000000048317 (PMC13124415; doi:10.1097/MD.0000000000048317)
Supplement: Supplementary file 6 [file medi-105-e48317-s006.pdf]

Supplemental Figure S1. Diagnostic plots of Mendelian randomization for the association between GCST90199628 and risk of COPD (forward and reverse directions)

Forward Mendelian randomization analyses：

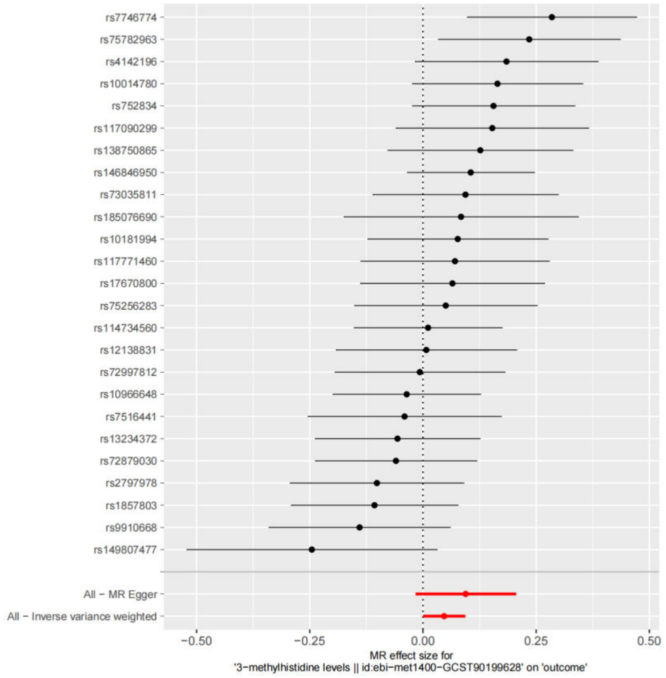

Forest plot for GCST90199628

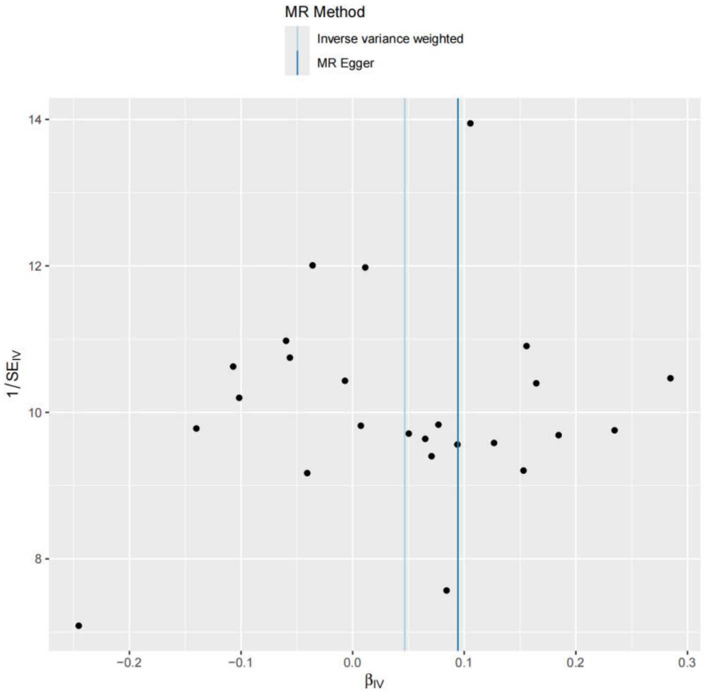

Funnel plot for GCST90199628

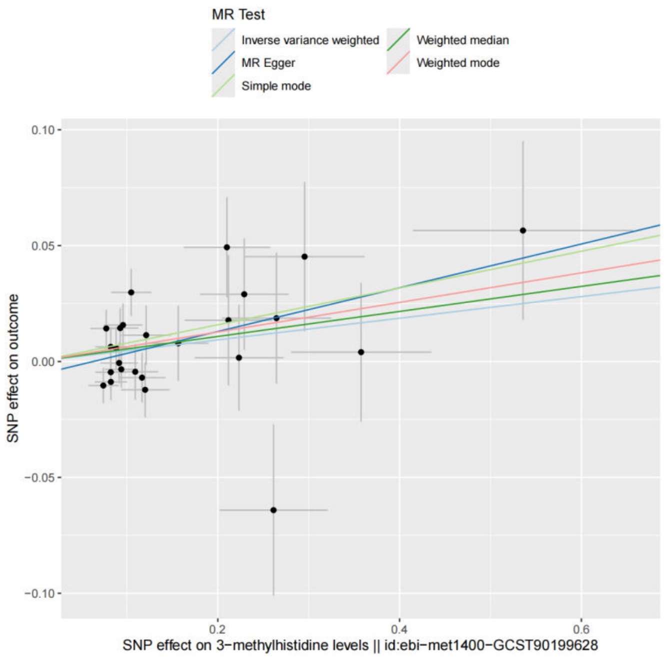

Scatter plot for GCST90199628

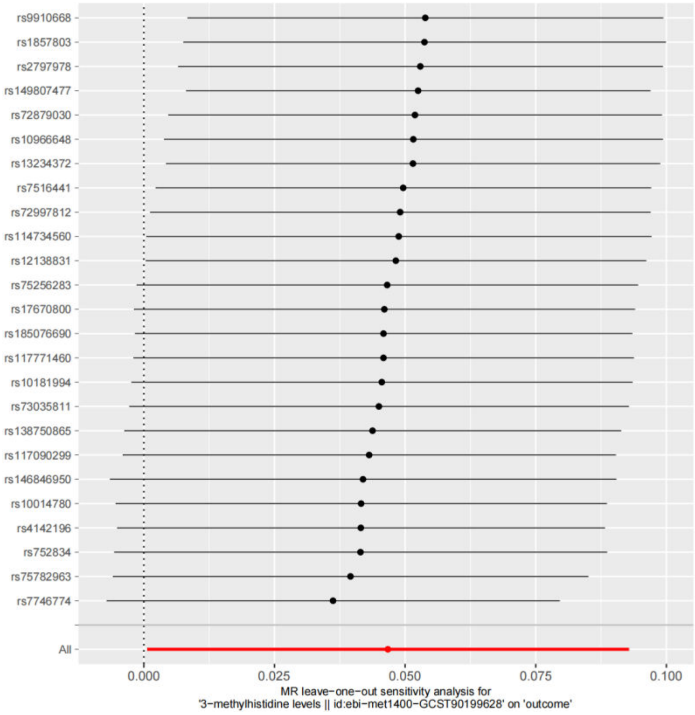

Leave-one-out sensitivity plot for GCST90199628

Reverse Mendelian randomization analyses：

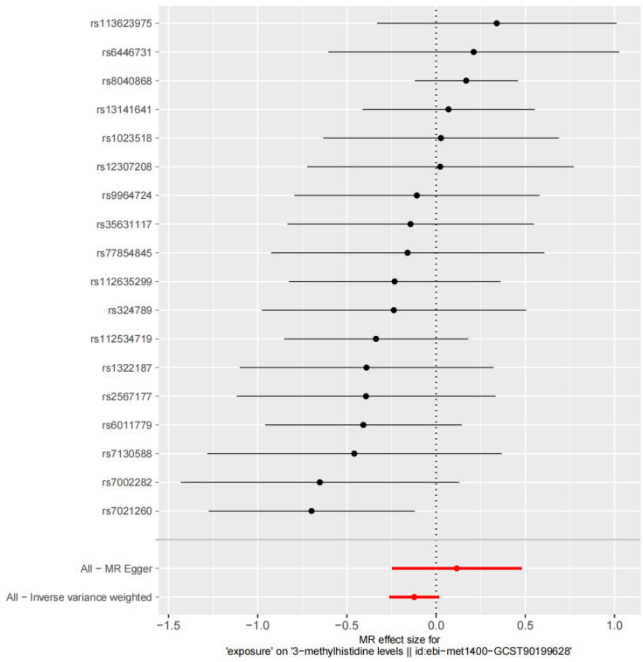

Forest plot for GCST90199628

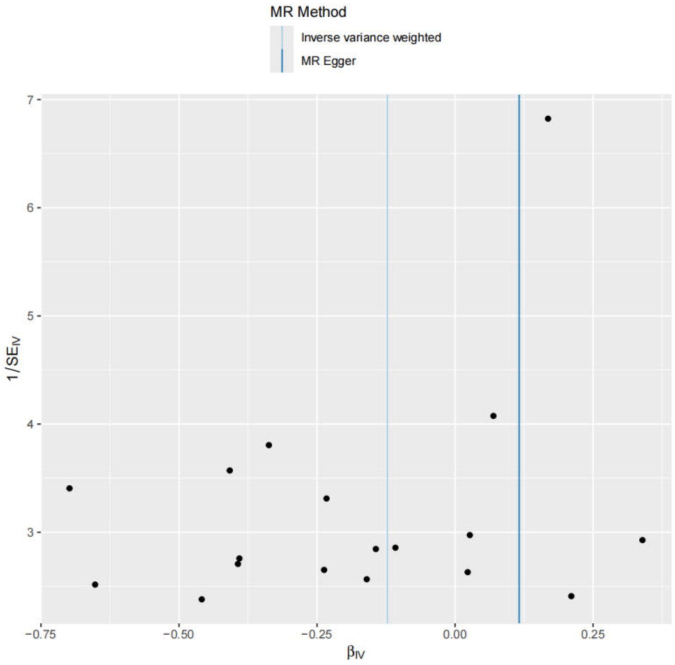

Funnel plot for GCST90199628

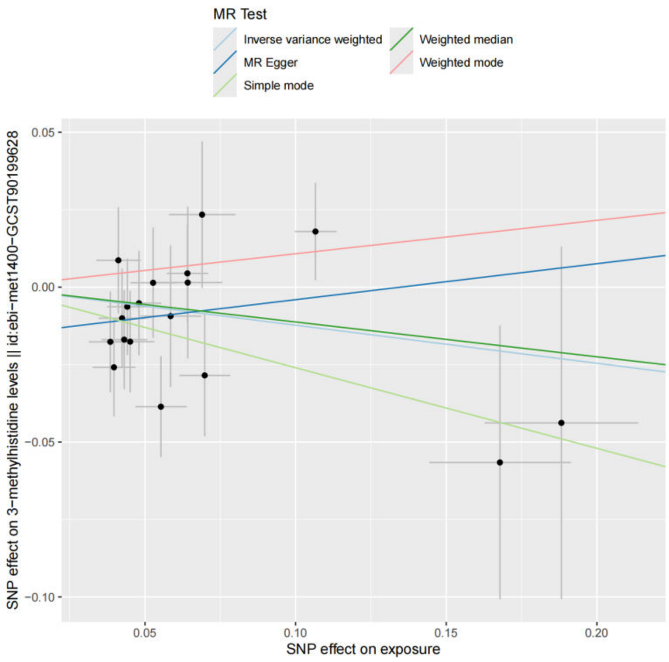

Scatter plot for GCST90199628

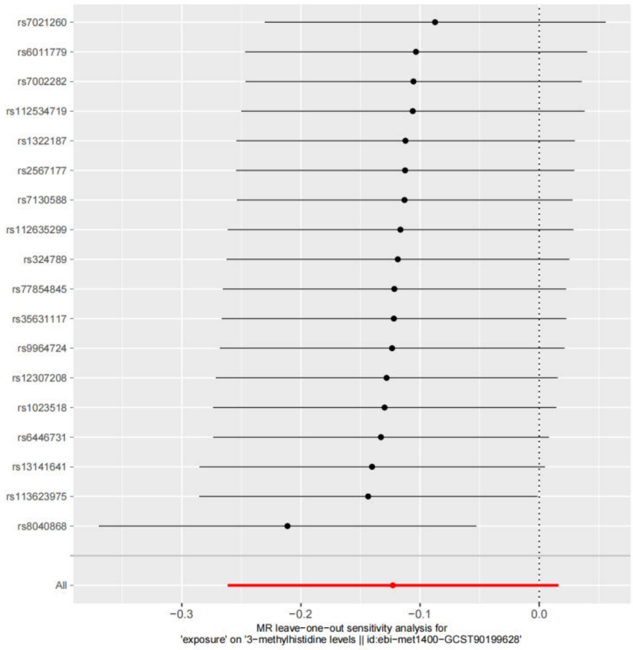

Leave-one-out sensitivity plot for GCST90199628

Supplemental Figure S2. Diagnostic plots of Mendelian randomization for the association between GCST90199624 and risk of asthma (forward and reverse directions)

Forward Mendelian randomization analyses：

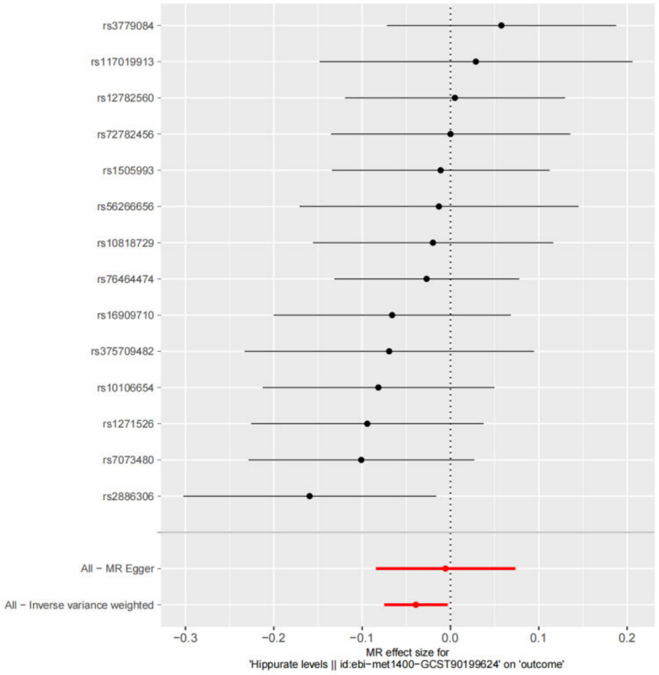

Forest plot for GCST90199624

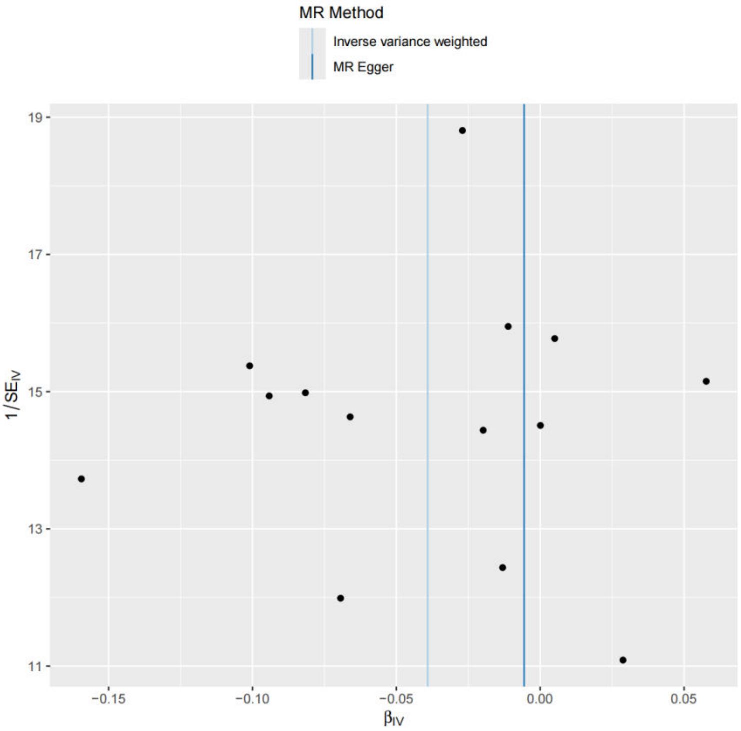

Funnel plot for GCST90199624

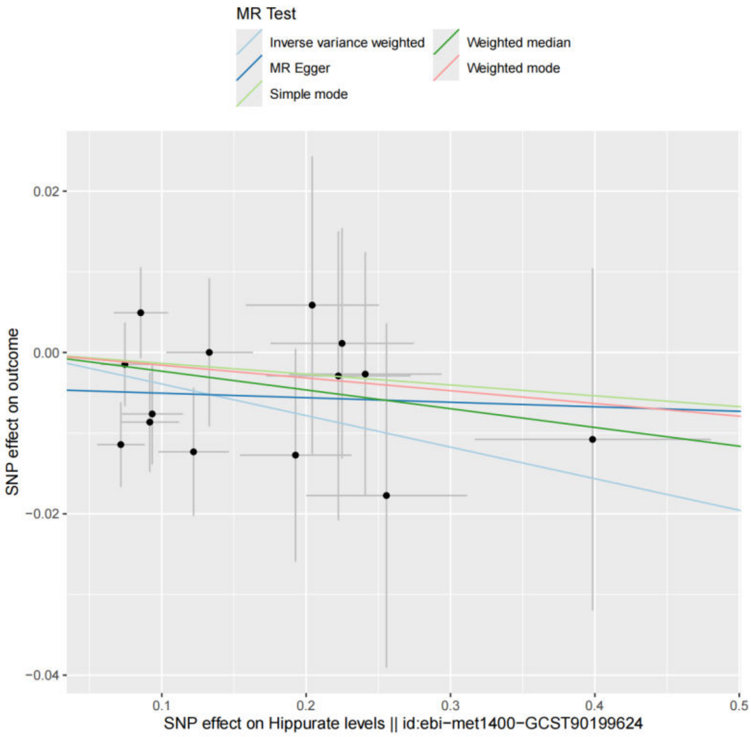

Scatter plot for GCST90199624

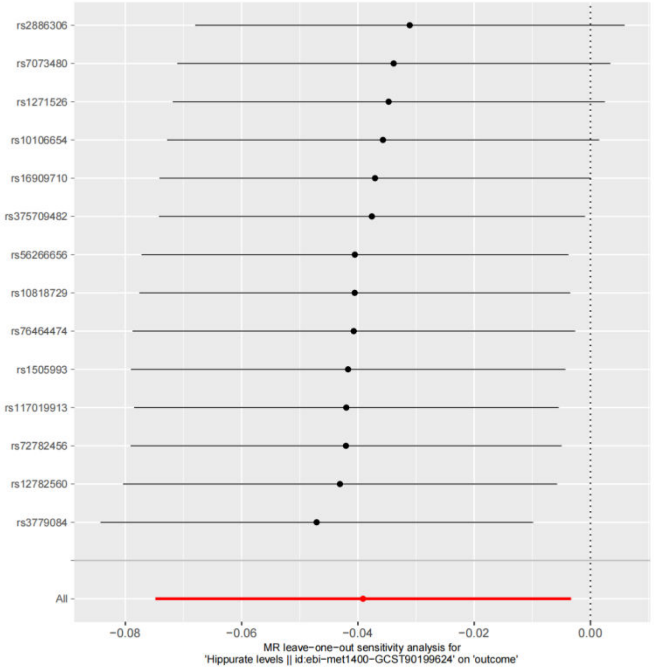

Leave-one-out sensitivity plot for GCST90199624

Reverse Mendelian randomization analyses：

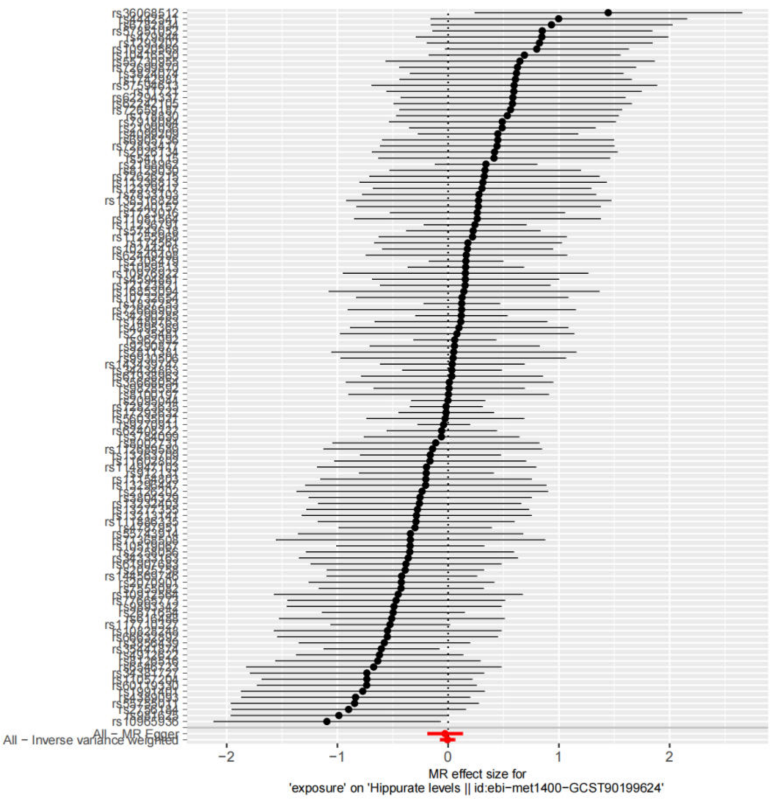

Forest plot for GCST90199624

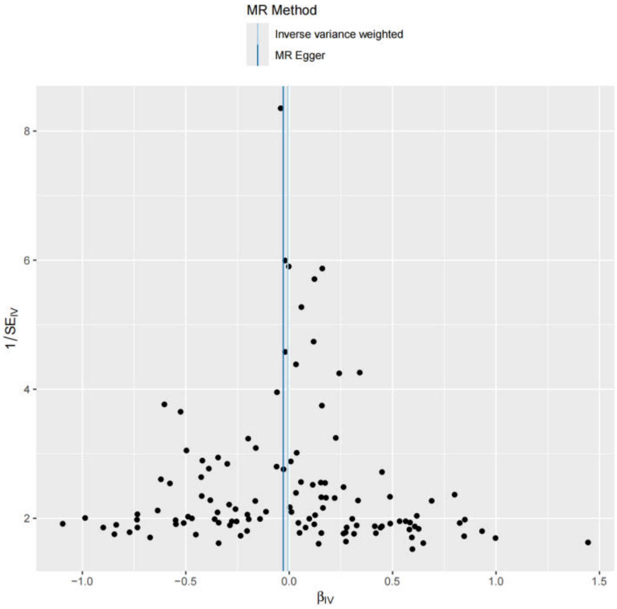

Funnel plot for GCST90199624

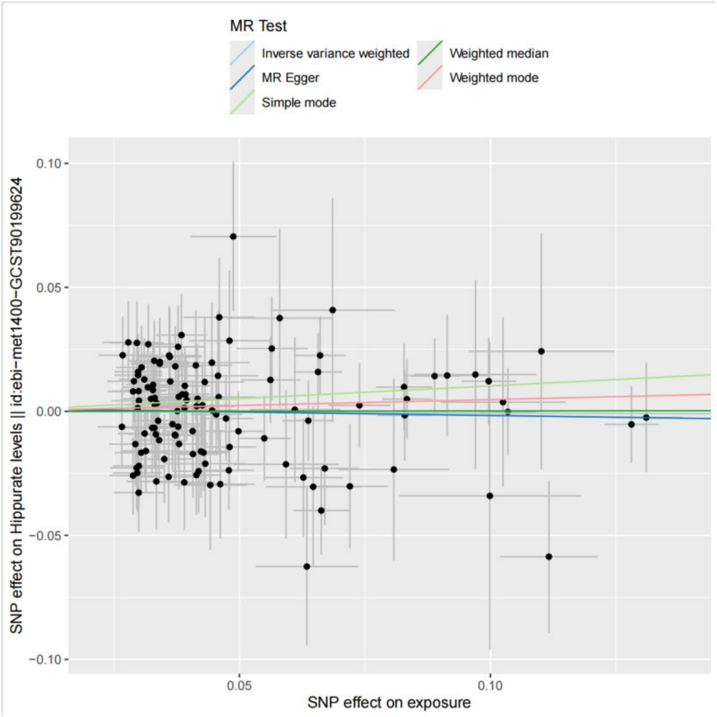

Scatter plot for GCST90199624

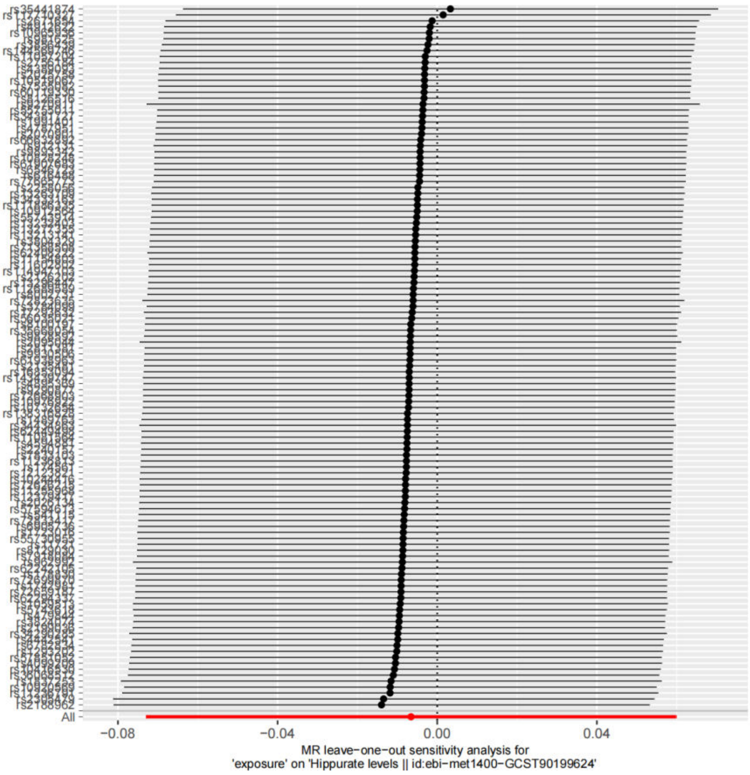

Leave-one-out sensitivity plot for GCST90199624

Supplemental Figure S3. Diagnostic plots of Mendelian randomization for the association between GCST90199640 and risk of IPF (forward and reverse directions)

Forward Mendelian randomization analyses：

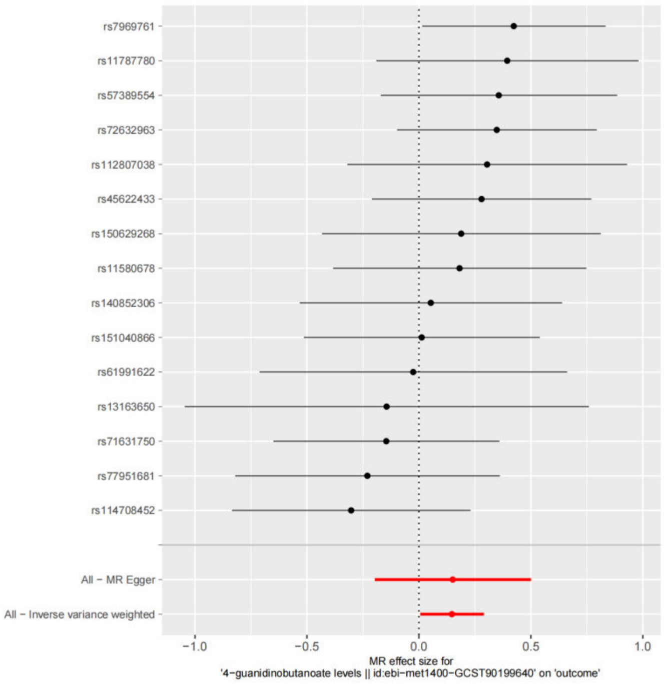

Forest plot for GCST90199640

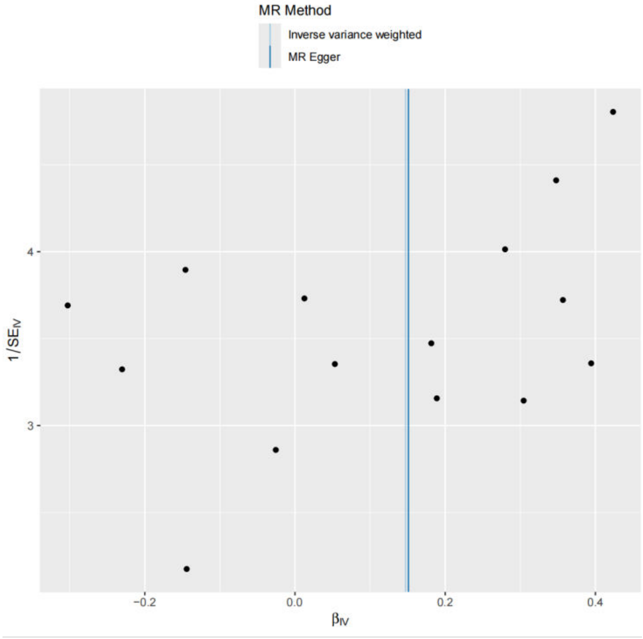

Funnel plot for GCST90199640

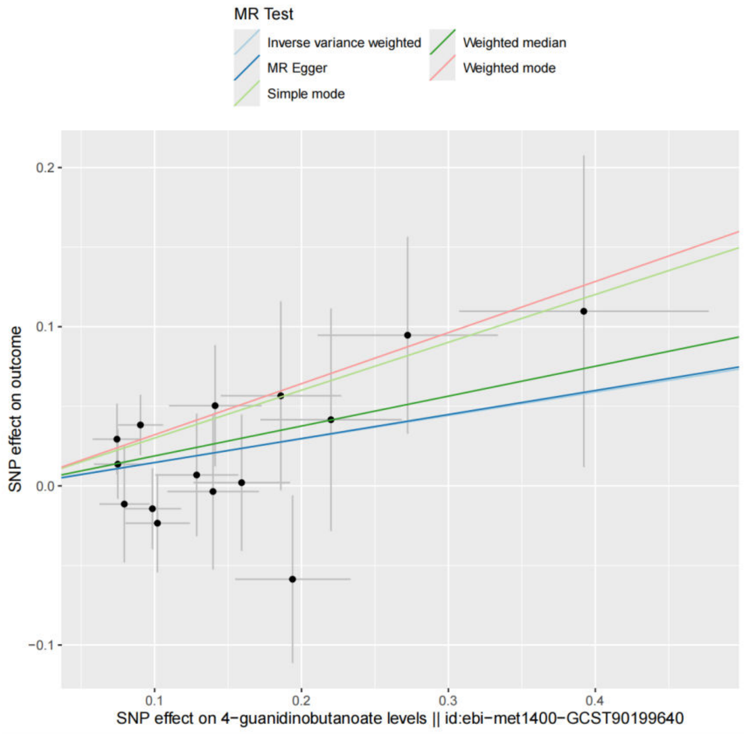

Scatter plot for GCST90199640

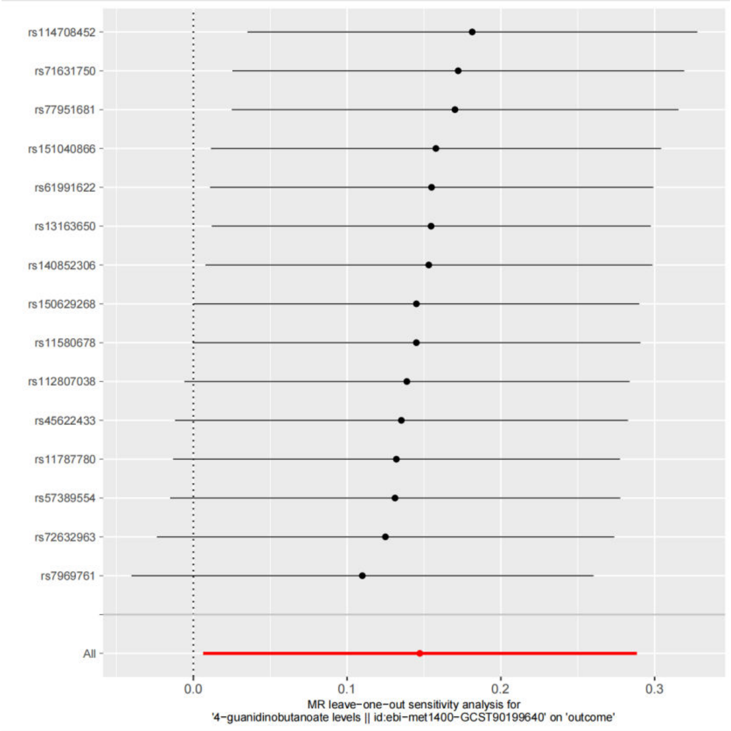

Leave-one-out sensitivity plot for GCST90199640

Reverse Mendelian randomization analyses：

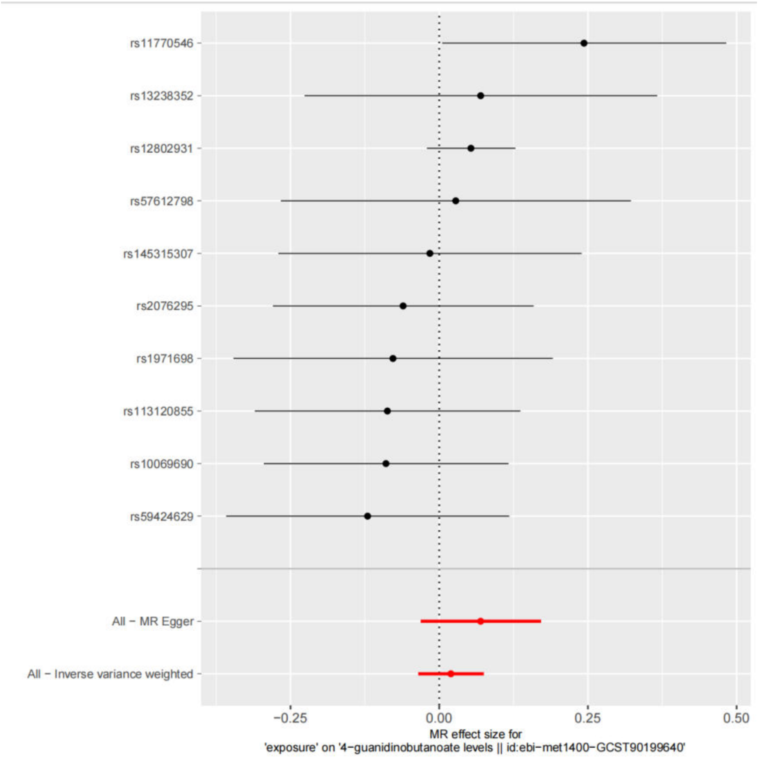

Forest plot for GCST90199640

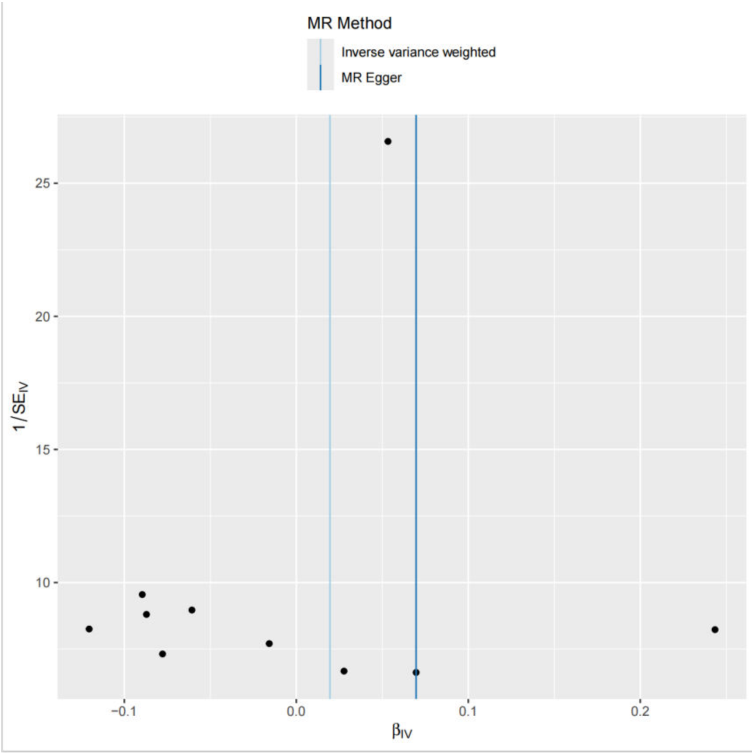

Funnel plot for GCST90199640

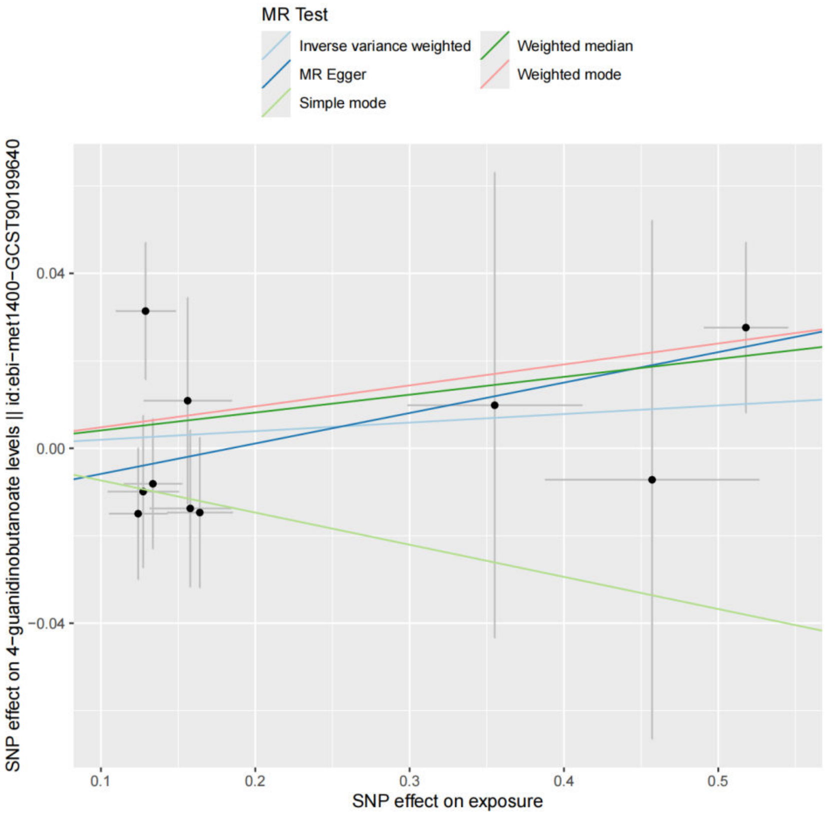

Scatter plot for GCST90199640

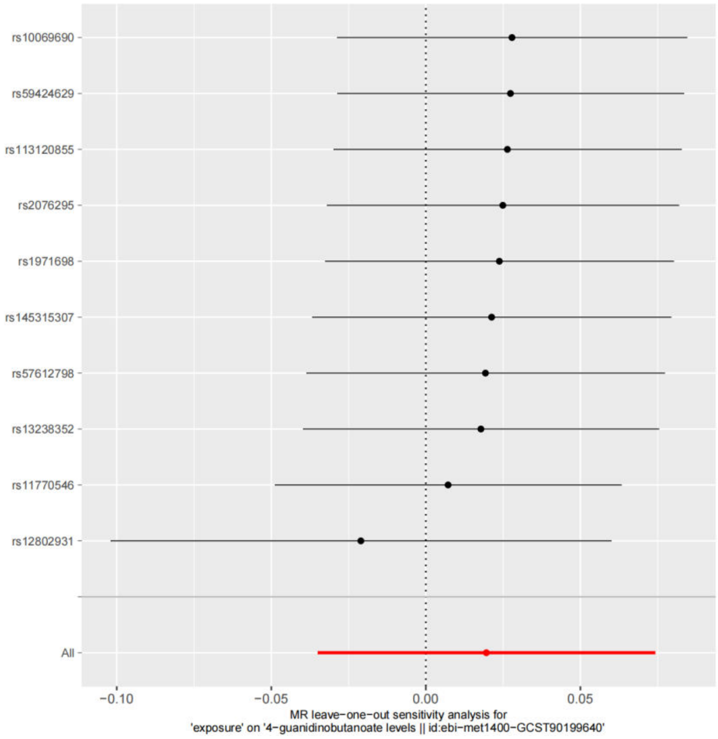

Leave-one-out sensitivity plot for GCST90199640

Supplemental Figure S4. Diagnostic plots of Mendelian randomization for the association between GCST90199623 and risk of sarcoidosis (forward and reverse directions)

Forward Mendelian randomization analyses：

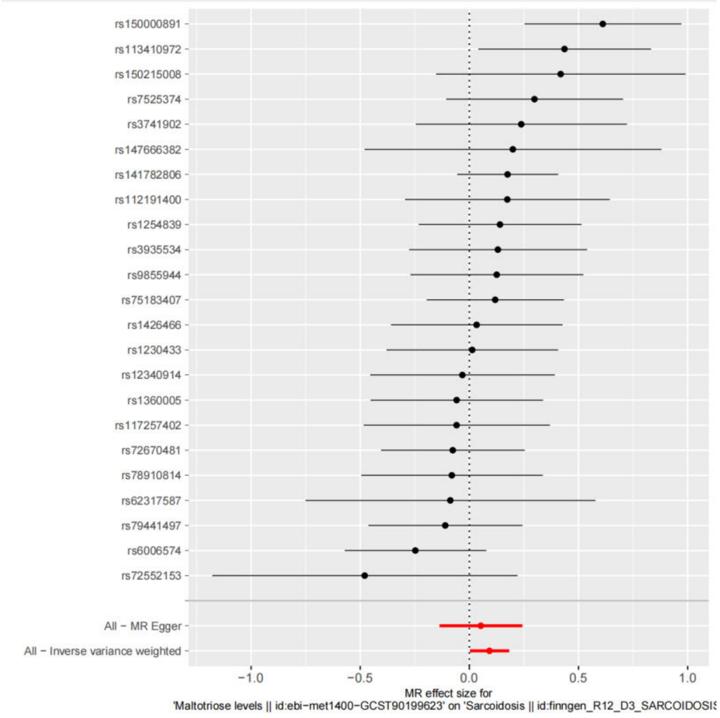

Forest plot for GCST90199623

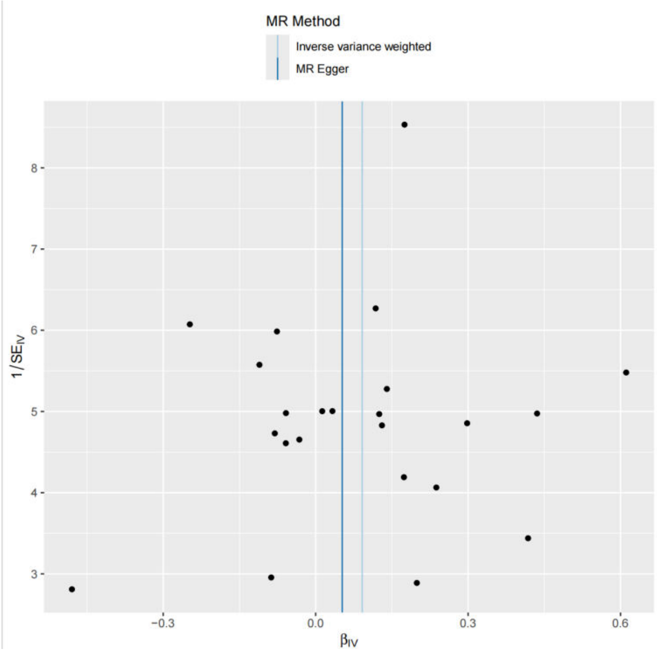

Funnel plot for GCST90199623

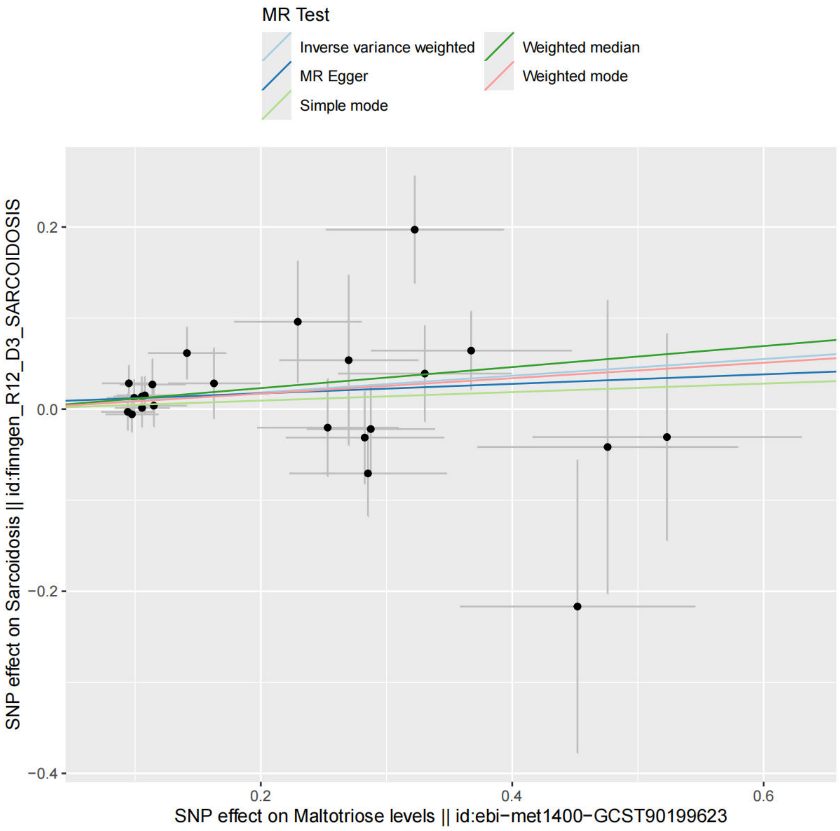

Scatter plot for GCST90199623

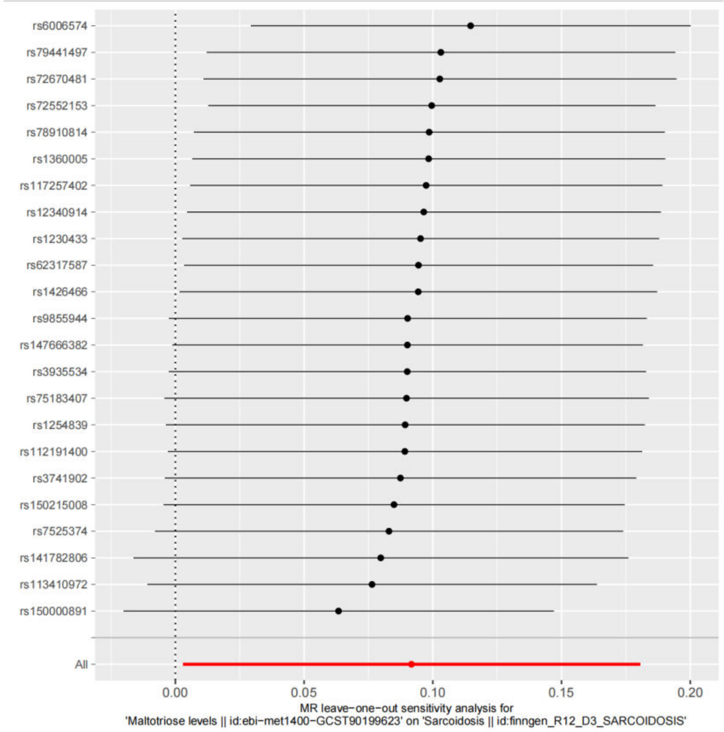

Leave-one-out sensitivity plot for GCST90199623

Reverse Mendelian randomization analyses：

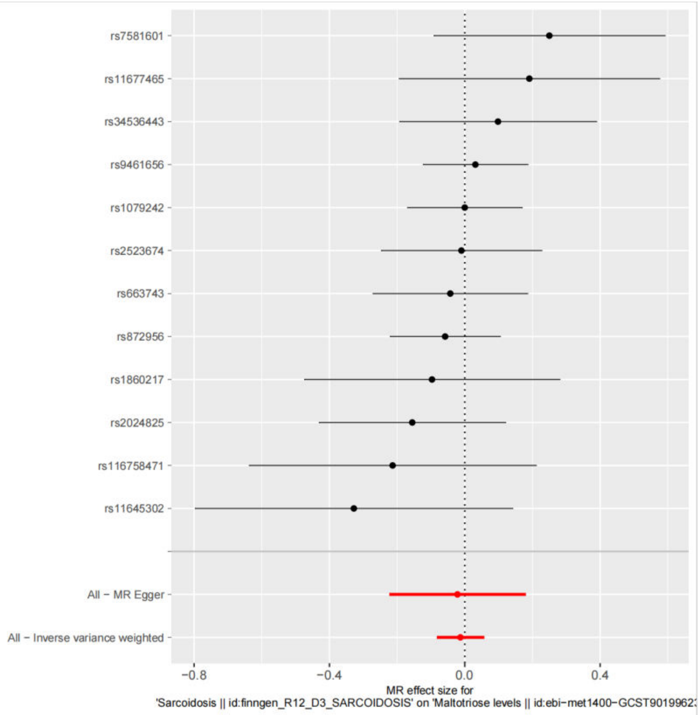

Forest plot for GCST90199623

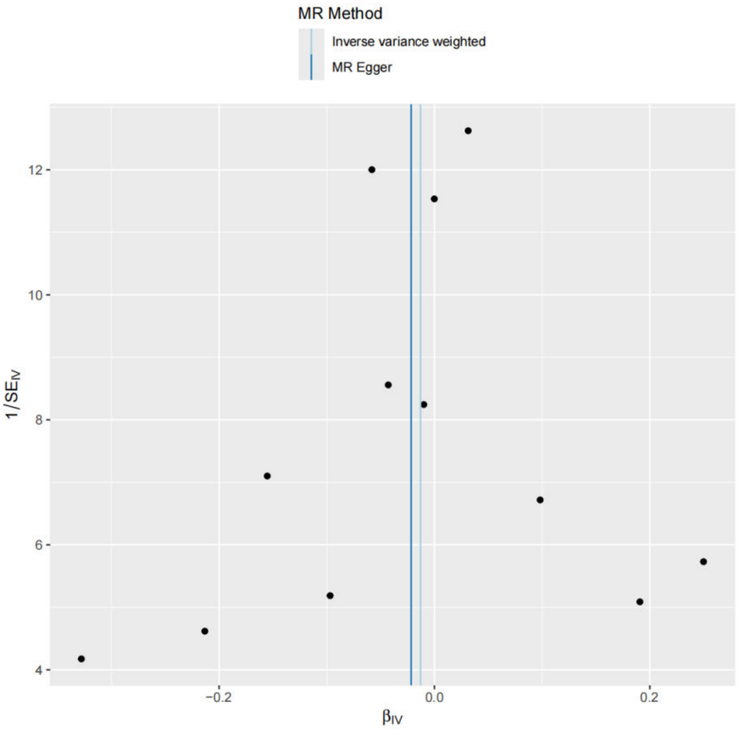

Funnel plot for GCST90199623

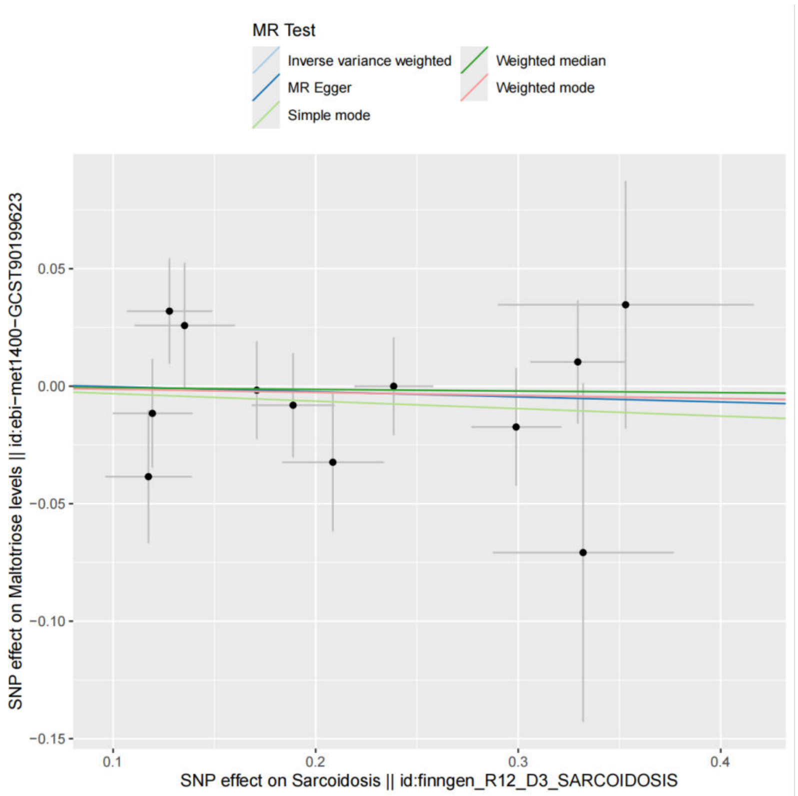

Scatter plot for GCST90199623

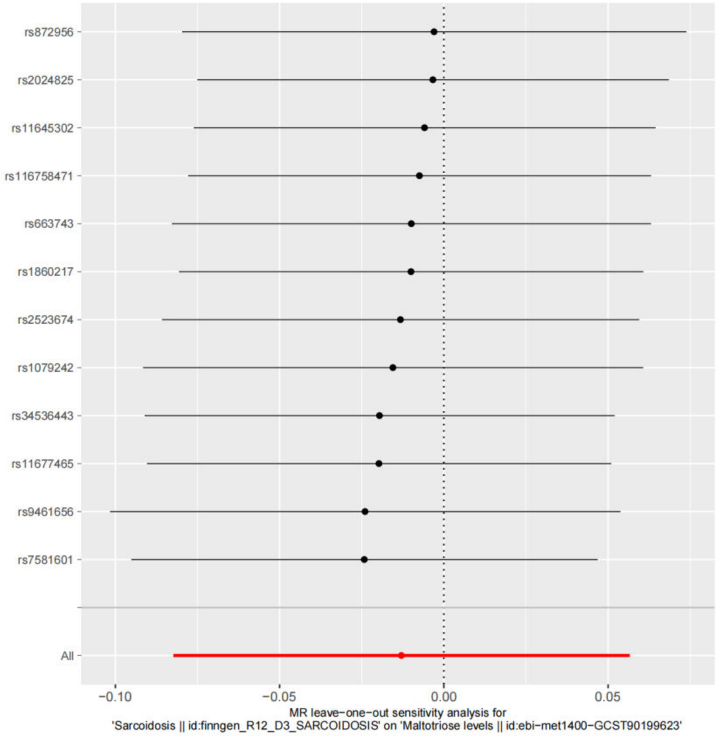

Leave-one-out sensitivity plot for GCST90199623

Supplemental Figure S5. Diagnostic plots of Mendelian randomization for the association between GCST90199661 and risk of pneumoconiosis (forward and reverse directions)

Forward Mendelian randomization analyses：

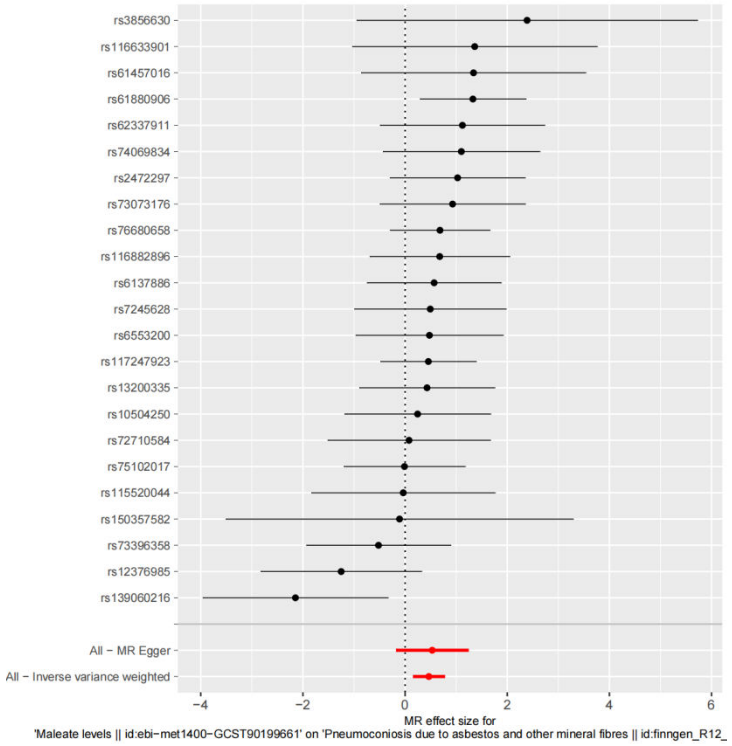

Forest plot for GCST90199661

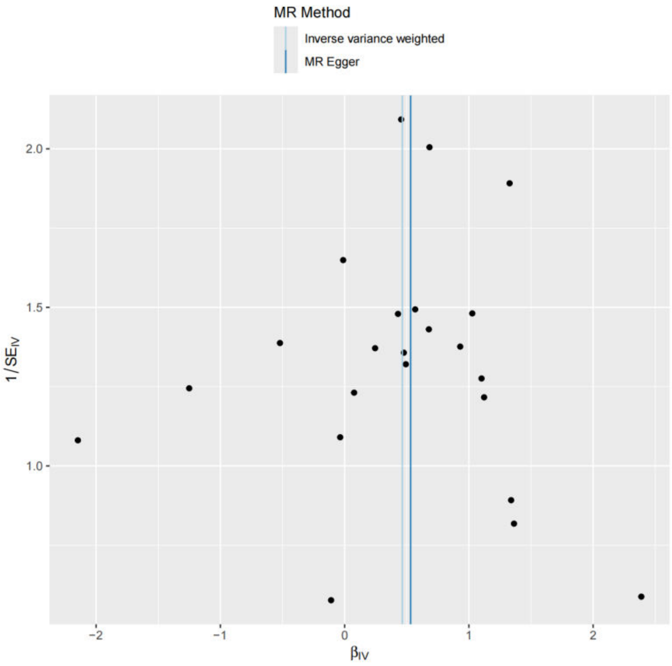

Funnel plot for GCST90199661

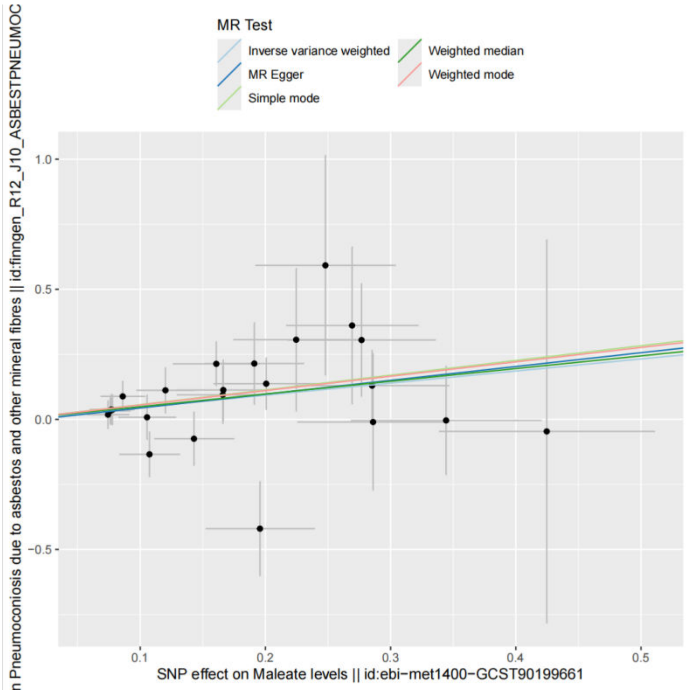

Scatter plot for GCST90199661

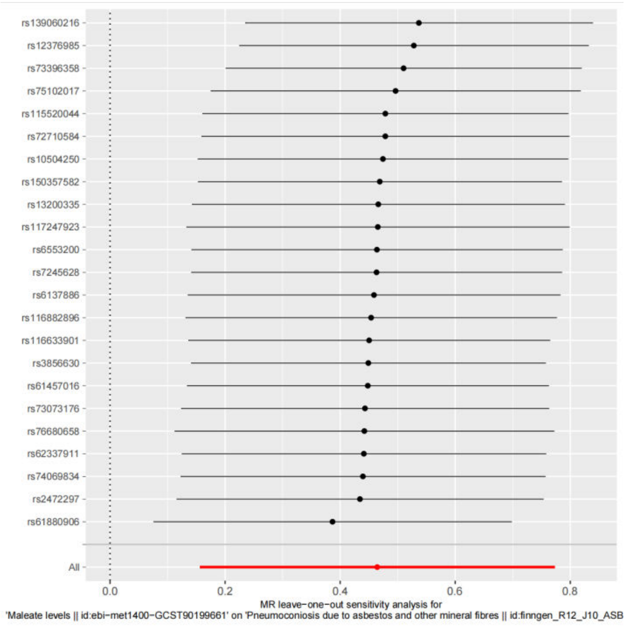

Leave-one-out sensitivity plot for GCST90199661

Reverse Mendelian randomization analyses：

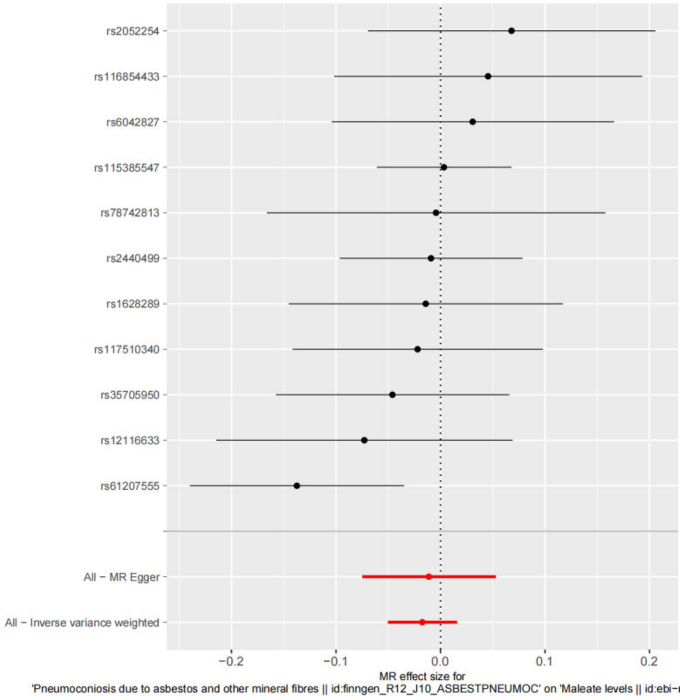

Forest plot for GCST90199661

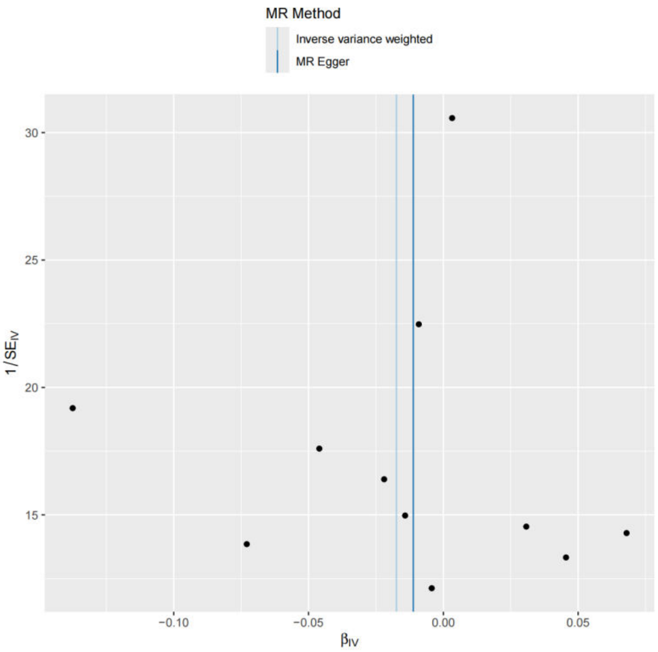

Funnel plot for GCST90199661

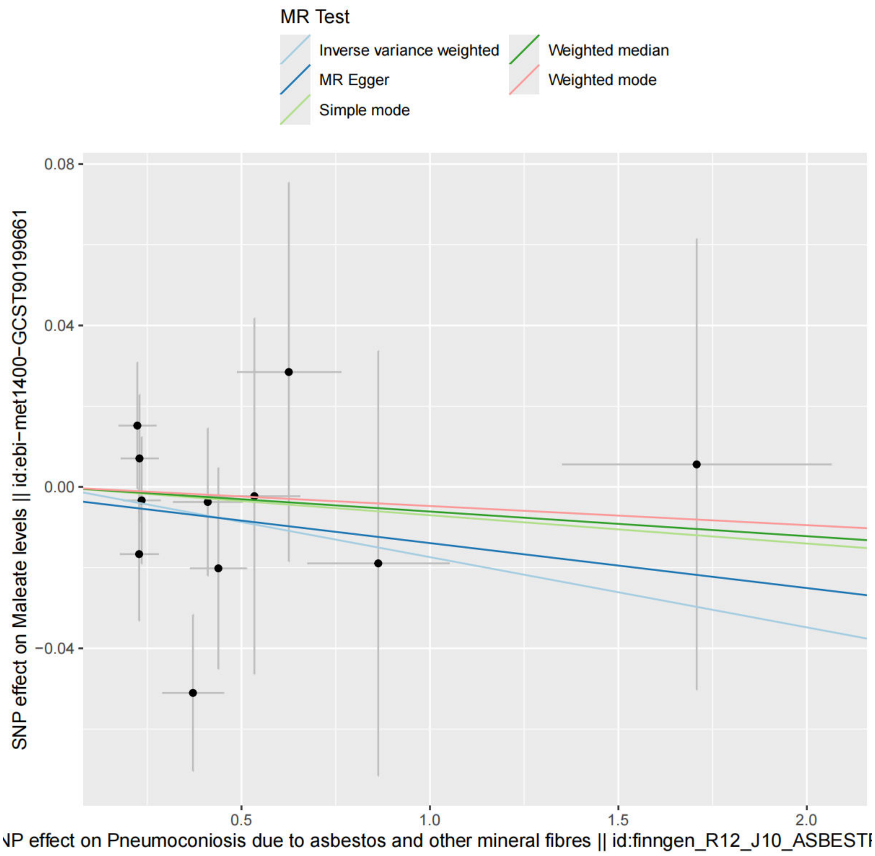

Scatter plot for GCST90199661

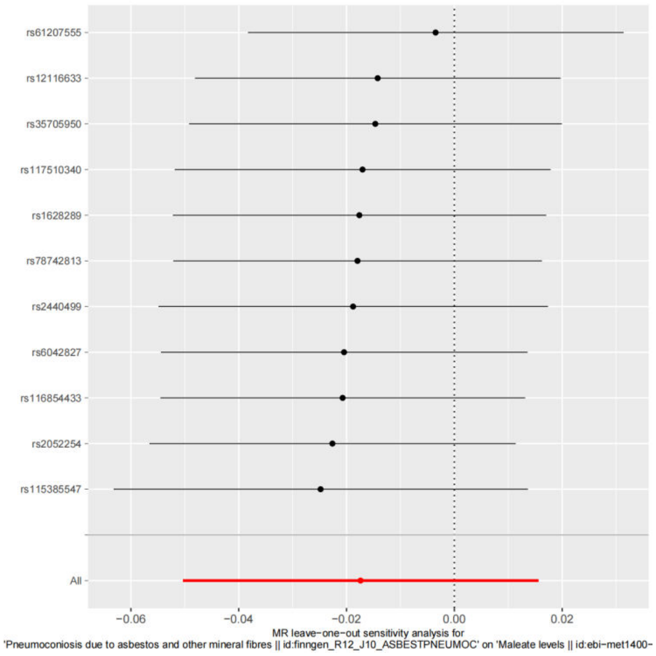

Leave-one-out sensitivity plot for GCST90199661
